# Importance of Cullin4 Ubiquitin Ligase in Malignant Pleural Mesothelioma

**DOI:** 10.3390/cancers12113460

**Published:** 2020-11-20

**Authors:** Mayura Meerang, Jessica Kreienbühl, Vanessa Orlowski, Seraina L. C. Müller, Michaela B. Kirschner, Isabelle Opitz

**Affiliations:** Department of Thoracic Surgery, University Hospital Zürich, 8091 Zürich, Switzerland; Jessica.Kreienbuehl@usz.ch (J.K.); Vanessa.Orlowski@usz.ch (V.O.); seraina.bomue@hotmail.com (S.L.C.M.); Michaela.Kirschner@usz.ch (M.B.K.)

**Keywords:** malignant pleural mesothelioma, NF2, ubiquitination, CUL4A, CUL4B, neddylation, pevonedistat

## Abstract

**Simple Summary:**

Malignant pleural mesothelioma (MPM) is an incurable and aggressive malignancy mainly caused by exposure to asbestos fibers. Survival outcomes following the standard of care treatment, including chemotherapy and surgery, remain dismal. Targeted treatments are shortcomings for MPM, as this tumor is driven primarily by a loss of tumor suppressor genes. In this study, we explored the importance of proteins that have been shown to be dysregulated in MPM as a consequence of tumor suppressor gene loss, CUL4A and CUL4B. We assessed their expression levels and identified their correlation with clinical outcomes of MPM. We also aimed to test the efficacy and mechanisms of the available treatments that target these proteins, pevonedistat using in vitro and in vivo models. Our data suggested that CUL4B might serve as a treatment target for MPM and revealed novel mechanism of pevonedistat in the MPM tumor microenvironment. This data may be useful for understanding its efficacy in patients from clinical trials.

**Abstract:**

Neurofibromatosis type 2 (NF2), the tumor suppressor frequently lost in malignant pleural mesothelioma (MPM), suppresses tumorigenesis in part by inhibiting the Cullin4 ubiquitin ligase (CUL4) complex in the nucleus. Here, we evaluated the importance of CUL4 in MPM progression and tested the efficacy of cullin inhibition by pevonedistat, a small molecule inhibiting cullin neddylation. CUL4 paralogs (CUL4A and CUL4B) were upregulated in MPM tumor specimens compared to nonmalignant pleural tissues. High gene and protein expressions of CUL4B was associated with a worse progression-free survival of MPM patients. Among 13 MPM cell lines tested, five (38%) were highly sensitive to pevonedistat (half maximal inhibitory concentration of cell survival IC_50_ < 0.5 µM). This remained true in a 3D spheroid culture. Pevonedistat treatment caused the accumulation of CDT1 and p21 in both sensitive and resistant cell lines. However, the treatment induced S/G2 cell cycle arrest and DNA rereplication predominantly in the sensitive cell lines. In an in vivo mouse model, the pevonedistat treatment significantly prolonged the survival of mice bearing both sensitive and resistant MPM tumors. Pevonedistat treatment reduced growth in sensitive tumors but increased apoptosis in resistant tumors. The mechanism in the resistant tumor model may be mediated by reduced macrophage infiltration, resulting from the suppression of macrophage chemotactic cytokines, C-C motif chemokine ligand 2 (CCL2), expression in tumor cells.

## 1. Introduction

Malignant pleural mesothelioma (MPM) arises from the malignant transformation of mesothelial cells lining the thoracic cavity. MPM is a rare but aggressive tumor commonly associated with the chronic inflammation of tissues exposed to asbestos fibers. The current standard first-line chemotherapy includes cis- or carboplatin and pemetrexed that holds only a 41% response rate [1]. Alternative treatment options are shortcomings for a tumor mainly driven by the loss of tumor-suppressor genes like MPM due to the lack of targetable oncogenic drivers. One of the most frequently lost tumor-suppressor gene in MPM is neurofibromatosis type 2 (*NF2*) encoding the protein Merlin. *NF2* is mutated in 19% of MPM, and the chromosomal region 22q containing this gene is commonly lost in MPM tumor tissues [2,3]. 

Merlin, a well-known tumor-suppressor protein, has been shown to regulate several oncogenic pathways [4], and its activity is tightly regulated by phosphorylation and localization [5]. A study by Li et al. suggested that only the unphosphorylated closed conformation of Merlin enters the nucleus to prevent tumorigenesis by inhibiting the Cullin4-RING E3 ubiquitin ligase complex (CRL4) [6]. Recently, our own research showed that low nuclear Merlin expression following chemotherapy was associated with worse survival outcomes of MPM patients [7]. Thus, CRL4 may serve as a potential pathway target for the treatment of MPM.

Cullin4 (CUL4) is a member of the cullin-RING ligase protein family. Cullins provide a core scaffold for ubiquitin ligase complex formation to catalyze the ubiquitination of target proteins for proteasomal degradation or activity changes [8,9]. Two CUL4 paralogs, CUL4A and CUL4B, share 82% similarity in protein sequences and have been shown to facilitate nucleotide excision repair and cell cycle progression [9]. Recent evidence revealed that the CUL4A gene (13q34) was amplified in MPM cell lines and overexpressed in 64% of MPM tumors [10]. In addition, CUL4A has also been shown to be amplified in breast cancer and was associated with a shorter overall and disease-free survival [8]. A study by Yang et al. using *cre*-inducible CUL4A expression demonstrated that CUL4A overexpression in the lung induced the development of adenocarcinoma in a mouse model [11]. Although CUL4B has been shown to be associated with the progression and tumorigenesis of other cancers, there is, so far, no study assessing whether CUL4B is important for MPM progression. In colon cancer, patients with CUL4B-positive tumors had a higher recurrence rate and shorter survival time compared to patients with CUL4B-negative tumors [12]. Transgenic mice overexpressing CUL4B in the liver exhibited increased hepatocyte proliferation and accelerated tumor development after chemical exposure [13]. 

Clear roles of CUL4 and other members of the cullin family in the regulation of cell growth and their association with the progression of various cancers suggested them as an attractive target molecule for the treatment of cancer. The conjugation of a ubiquitin-like protein, NEDD8 (neural precursor cell expressed, developmentally downregulated 8), stimulates the activity of cullins by inducing conformational changes to facilitate a ubiquitin transfer [14]. A small molecule MLN-4924 (pevonedistat) inhibits cullins by inhibiting protein neddylation. By binding covalently to the active site of the NEDD8-activating enzyme (NAE), the pevonedistat-NAE adduct cannot be processed any further in the NEDD8 pathway [15]. Pevonedistat demonstrated antineoplastic activity by inducing the apoptosis and scenence of cancer cells [16]. Pevonedistat has entered several Phase I and II clinical trials for hematologic malignancies and solid tumors, including MPM.

We hypothesized that altered ubiquitination events resulting from Merlin loss or CUL4A/B gain of function is one of the drivers of MPM progression. In this study, we assessed the importance of both CUL4 paralogs in MPM and tested whether the pharmacological inhibition of cullins by pevonedistat is effective for MPM. Although pevonedistat has been tested in MPM in the context of *NF2* loss and now is being tested in clinical trials for MPM, there has not been a mechanistic detail explaining the treatment efficacy in vivo and possible effects on the tumor microenvironment. Here, we employed a panel of MPM cell lines to assess the efficacy and mechanisms of pevonedistat in in vitro 2D and 3D models prior to confirming in vivo using a mouse model in which the tumors develop on the mesothelial tissues of the peritoneal cavity, providing a more relevant tumor microenvironment. We also assessed the long-term outcome of the pevonedistat treatment by monitoring the overall survival after the treatment.

## 2. Results

### 2.1. CUL4A and CUL4B Expression Was Elevated in a Subset of MPM Patients, and High CUL4B Expression Was Associated with a Poor Outcome

**Figure 1:**
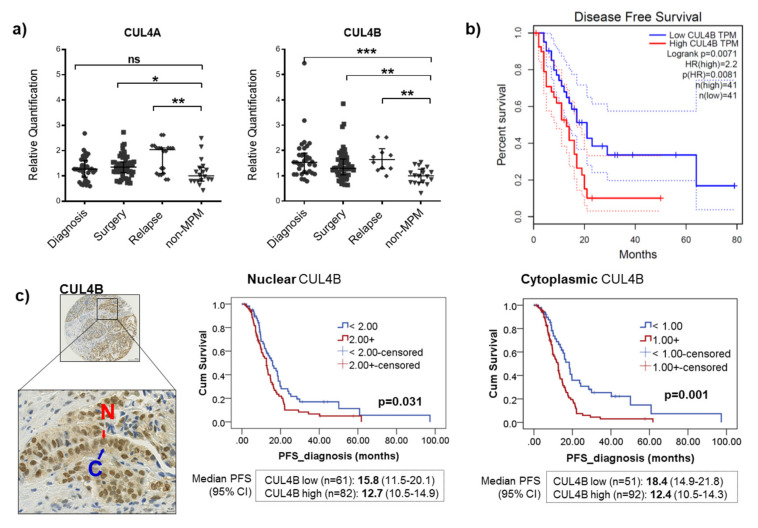
Significance of CUL4A and CUL4B expressions in malignant pleural mesothelioma (MPM). (**a**) qPCR analysis showing the upregulation of CUL4A and CUL4B gene expressions in a subset of MPM tissues collected at various time points (diagnosis (*n* = 34), surgery (*n* =50) and relapse (*n* = 10)) compared to non-MPM tissues (*n* = 18). We used β-actin as a reference gene and the median threshold cycle difference between CUL4A or CUL4B compared to β-actin (∆Ct) of the non-MPM group as a reference value for relative quantification by the 2^−∆∆Ct^ method. The bars show medians with interquartile ranges. Mann Whitney test, ns, not significant; * *p* < 0.05, ** *p* < 0.001 and *** *p* < 0.0001. (**b**) Gene expression dataset from TCGA showing an association between high CUL4B expression with shorter disease-free survival (*n* = 82). (**c**) Example of immunohistochemical staining of CUL4B showing protein localization predominantly in the nucleus (N); cytoplasmic (C) expression is present but at a lower extent. High protein expression of CUL4B in both nucleus and cytoplasm in this patient cohort (*n* = 143) is associated with shorter PFS. The data were dichotomized according to the median gene or protein expression of the patient cohort for Kaplan-Meier curves. We used a log-rank test for the comparison of survival differences. PFS, progression-free survival and CI, confidence interval.

We assessed the gene expression of CUL4A and CUL4B in 94 tumor tissues collected from MPM patients at diagnosis, surgery or relapse by quantitative real-time PCR (qPCR). Non-MPM samples are tissue samples collected from patients with chronic inflammation of the pleura for whom the final diagnosis was negative for MPM. CUL4A, and, more significantly, CUL4B expression were elevated in a subset of MPM tumors from all time points (Figure 1a). We did not find any correlation between CUL4A and CUL4B expression with the progression-free survival (PFS) and overall survival (OS) of patients in this cohort (data not shown), most probably due to the small sample size. Gene expression data from the publicly available TCGA database (MESO dataset) showed that a high CUL4B gene expression was associated with a shorter disease-free survival of patients (Figure 1b), while there was no association between the CUL4A expression with survival outcomes (data not shown) [17]. Accordingly, the protein expression by immunohistochemistry on a tissue microarray of our MPM patient cohort showed that high CUL4B protein expression was associated with a short PFS of the patients (Figure 1c). In contrast, on the protein expression level, we found that high cytoplasmic CUL4A expression was associated longer PFS (*p* = 0.01). The details of the patient cohort for the tissue microarray are provided in Table S1. There were no correlations between the CUL4A and CUL4B protein expressions with histological subtypes of MPM.

### 2.2. CU4LA and CUL4B Are Overexpressed in MPM Cell Lines, and a Subset of Them Were Susceptible to the Inhibition of NEDD8 Activating Enzyme (NAE) by Pevonedistat

**Figure 2:**
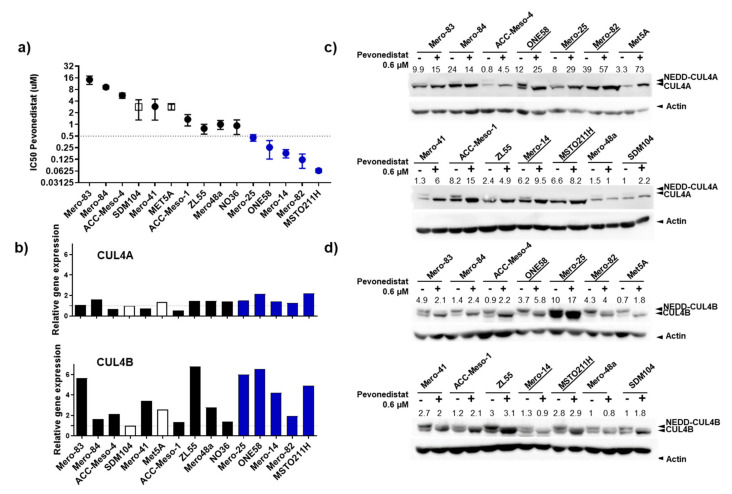
In vitro response of MPM cell lines to pevonedistat. (**a**) IC_50_ showing the pevonedistat concentration (µM) required for the inhibition of 50% cell survival after 3 days of drug exposure (3-(4,5-dimethylthiazol-2-yl)2,5-diphenyltetrazolium bromide (MTT) assay). The graph depicts the mean IC_50_ from at least two independent experiments from each cell line. (**b**) Gene expressions of CUL4A and CUL4B in MPM cell lines normalized to the expression of the nonmalignant mesothelial cell line SDM104. (**c**,**d**) Western blot analysis showing protein expressions of CUL4A and CUL4B in MPM cell lines and nonmalignant mesothelial cell lines (Met5A and SDM104). The upper band (see arrows) of CUL4A and CUL4B represent the neddylated form of both proteins. Treatment with 0.6-µM pevonedistat for 24 h sufficiently inhibited the neddylation of CUL4A and CUL4B in most of the cell lines. The number above each line of the blots represents the protein expression (ratio densitometry of the bands normalized with β-actin) relative to untreated SDM104. Underlined are the cell lines sensitive to the neural precursor cell expressed, developmentally downregulated (NEDD) inhibition by pevonedistat.

We employed a panel of 13 MPM cell lines and two nonmalignant mesothelial cells (Met5A and SDM104 [18]) for an assessment of MPM sensitivity and response to pevonedistat treatment. While nonmalignant mesothelial cells are relatively resistant to the treatment ((half maximal inhibitory concentration of cell survival (IC_50_), 2.4 and 2.7 µM for Met5A and SDM104, respectively), a subset (*n* = 5, 38%) of MPM cell lines are very sensitive to the treatment with pevonedistat (IC_50_ < 0.5 µM) (Figure 2a and Table S2). Examples of IC_50_ curves are provided in Figure S1. qPCR analysis showed that 67% of MPM cell lines overexpressed CUL4A (up to ~two-fold), while all overexpressed CUL4B (up to ~six-fold) compared to SDM104 (Figure 2b). A protein expression analysis showed that 92% of the MPM cell lines overexpressed the CUL4A protein at a much higher extent compared to the gene expression (up to ~39-fold, Figure 2c), while 75% overexpressed CUL4B with a similar extent compared to the gene expression (up to ~10.2-fold, Figure 2d). There was a significant positive correlation between CUL4B mRNA (Figure 2b) and protein levels of the untreated condition shown in Figure 2d (r = 0.57, *p* = 0.04), while there was no positive correlation for CUL4A. The Western blot analysis shows that 0.6-µM pevonedistat efficiently inhibited CUL4A and CUL4B neddylation (upper bands) in most of the cell lines (Figure 2c,d). There was no correlation between CUL4A and CUL4B expression with the sensitivity to pevonedistat. 

### 2.3. Increased Accumulation of Cells with DNA Rereplication in Pevonedistat-Sensitive Cell Lines

**Figure 3:**
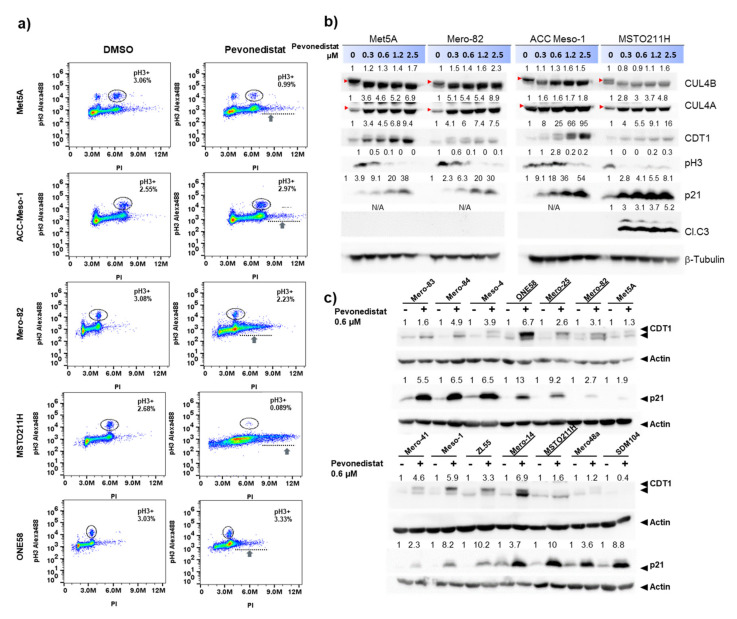
Effects of pevonedistat treatment on cell cycle progression, p21 and CDT1 accumulation. (**a**) Cell cycle analysis by double staining with (propidium iodide) PI and pH3 showing an accumulation of cells in the S or G2 phase of the cell cycle predominantly in sensitive cells (Mero-82, MSTO211H and ONE58) treated with 0.3-µM pevonedistat for 24 h compared to control (treated only with dimethylsulfoxide (DMSO)). We noted a drastically increased accumulation of cells containing >4N DNA after the treatment, predominantly in the sensitive cell lines, without increased accumulation of cells undergoing mitosis (pH3^+^, circles). (**b**) Cells were treated with an increasing concentration of pevonedistat for 24 h and subjected to Western blot analysis for the indicated proteins. Number above each line of the blots represents protein expressions (ratio densitometry of the bands normalized with β-actin) relative to no pevonedistat treatment. Arrows indicate neddylated CUL4A or CUL4B. (**c**) A panel of MPM cell lines treated with DMSO or 0.6-µM pevonedistat for 24 h shows an accumulation of CDT1 and p21 in both sensitive (underlined) and resistant cell lines. Number above each line of the blots represents protein expressions (ratio densitometry of the bands normalized with β-actin) relative to no pevonedistat treatment.

We next investigated the effect of pevonedistat treatment on the cell cycle. We observed increased numbers of cells in the S and G2/M phases or cells containing >4N DNA in all the cell lines tested (Figure S2 and Figure 3a). This is, however, more pronounced in sensitive cell lines (Mero-82, MSTO211H and ONE58). An increased number of cells in the sub-G1 population was only detected in the sensitive MSTO211H cells after 24-h treatment (Figure S2). We therefore stained the cells with phospho-histone H3 (ser10) (pH3) antibody to test whether the >4N population enters mitosis. The staining revealed that >4N cells are negative for pH3 (Figure 3a), suggesting DNA rereplication in this cell population. These data suggested that the effects of pevonedistat in sensitive cell lines is mediated primarily through cell cycle arrest and increased DNA rereplication. These are known effects of pevonedistat mediated by an accumulation of chromatin licensing and DNA replication factor 1, CDT1 and cyclin-dependent kinase inhibitor, p21 [19,20]. CUL1 and CUL4 are known to involve in the degradation of CDT1 and p21 during cell cycle progression. The Western blot analysis showed increased CDT1 and p21 accumulation in all cell lines, and there was no difference in the extent of CDT1 and p21 accumulation comparing sensitive and resistant cell lines (Figure 3b,c). All cell lines showed reduced proliferation with 0.3-µM pevonedistat (according to pH3 staining), but the growth of ACC-Meso-1 was affected starting from 1.2-µM pevonedistat (Figure 3b). Only the MSTO211H cell line showed increased apoptosis (based on cleaved caspase-3 (Cl.C3) staining) following the treatment. Pevonedistat inhibited CUL4A and CUL4B neddylation (upper bands) effectively in both sensitive (Mero-82 and MSTO211H) and resistant cell lines (Met5A and ACC-Meso-1) (Figure 3b). In conclusion, although pevonedistat efficiently inhibited CUL4 neddylation and the degradation of CDT1 and p21 in both sensitive and resistant cell lines, the extent of DNA replication is more pronounced in the sensitive cell lines.

### 2.4. Three-Dimentional Culture Confirmed Differential Sensitivity of MPM Cell Lines to Pevonedistat

**Figure 4:**
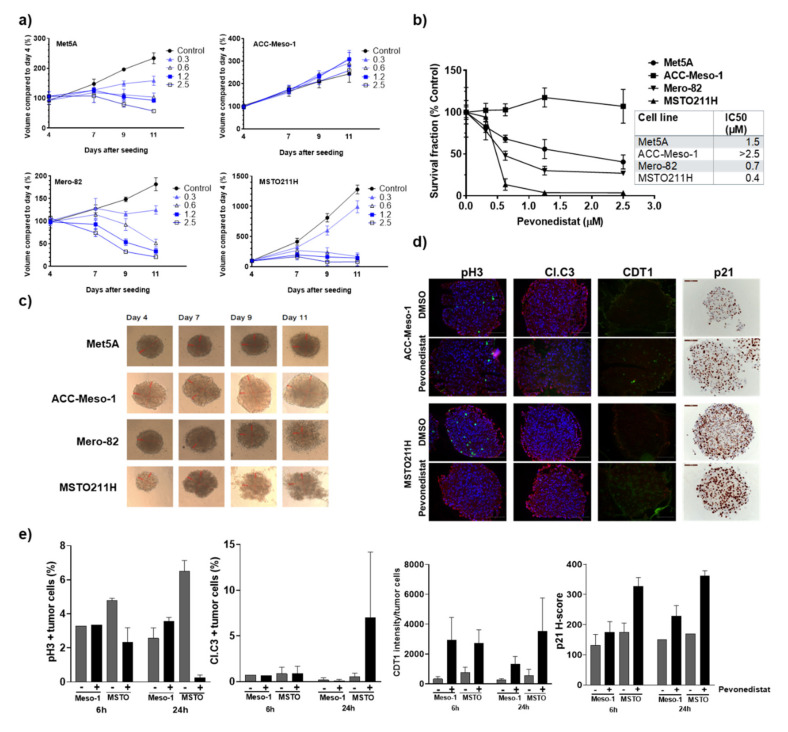
Response of MPM cell lines grown in 3D culture to pevonedistat. (**a**) Cells grown in multicellular spheroids were treated on day 4 and day 7 after seeding with the indicated increasing concentration of pevonedistat (µM). To monitor the drug efficacy, we measured the spheroid volume on days 4, 7, 9 and 11. The graphs were plotted as a percentage of the sphere volume compared to day 4. Pevonedistat effectively inhibited the growth of Met5A, Mero-82 and MSTO211H without affecting ACC-Meso-1 at any concentration tested. (**b**) The IC_50_ of pevonedistat in the inhibition of the spheroid cell survival, measured by the acid phosphatase (APH) assay on day 11, confirmed that Mero-82 and MSTO211H are sensitive to the treatment while ACC-Meso-1 is resistant. (**c**) Representative images of spheroids treated with 0.6-µM pevonedistat. The presence of dead cells was visible in Mero-82 and MSTO211H. Red lines are the measurements of the spheroid diameters. (**d**) Immunofluorescent staining of pH3, cleaved caspase-3 (Cl.C3), CDT1 (green) and immunohistochemical staining of p21 (brown) in spheroids after 24 h treatment with 0.6-µM pevonedistat. Fluorescent stainings were co-stained with DNA staining with 4′,6-Diamidino-2-Phenylindole (DAPI, blue) and pan-cytokeratin (red). DAPI signal was omitted from CDT1 staining for better visualization of the CDT1 signal. Scale bars represent 100 µm. (**e**) Quantification of the staining showed the accumulation of CDT1 and p21 in both cell lines after treatment, but only affected the proliferation (pH3) and induced apoptosis (Cl.C3) in MSTO211H.

Cancer cells grown in three-dimensional (3D) are shown to better recapitulate the drug resistance observed in patients than 2D cell culture experiments. Therefore, we selected four cell lines (resistant ACC-Meso-1, nonmalignant mesothelial cell Met5A and sensitive cell lines Mero-82 and MSTO211H) to confirm their sensitivity to pevonedistat in 3D culture. While the IC_50_ of pevonedistat was reduced in 3D culture of Met5A (IC_50_ 2D vs. 3D culture; 2.8 vs. 1.5 µM), all MPM cell lines showed an increased IC_50_ of at least five times compared to 2D culture (IC_50_ 2D vs. 3D culture; ACC-Meso-1 1.4 vs. >2.5 µM, Mero-82 0.12 vs. 0.7 µM and MSTO211H 0.06 vs. 0.4 µM). Similar to the results obtained from the 2D culture, these cells still exhibited differential sensitivity to pevonedistat when measured by spheroid growth (Figure 4a) or cell survival at end of the treatment (Figure 4b). Treatment with 0.6-µM pevonedistat induced cell death and the dissociation of cells from spheroids in Mero-82 and MSTO211H, while it only moderately affected Met5A growth without visible cell death and showed no effect on ACC-Meso-1 (Figure 4c). We also treated spheroids with cisplatin, a standard of care chemotherapy for MPM, to test whether the drug resistance is specific for pevonedistat or a general characteristic of each cell line. There is a switch in the resistant pattern, with pevonedistat-sensitive Mero-82 being relatively resistant to cisplatin while pevonedistat-resistant Met5A are sensitive to the treatment with cisplatin (Figures S3 and S4). We therefore concluded that this is rather a drug-specific resistance mechanism. We then analyzed the proliferation (pH3) and apoptosis (Cl.C3), CDT1 and p21 levels in ACC-Meso-1 and MSTO211H spheroids (Figure 4d,e). We clearly observed a reduced proliferation of MSTO211H as early as six h after the treatment, while ACC-Meso-1 was not affected up to 24 h after the treatment. Treatment-induced apoptosis can be observed in MSTO211H at a 24 h post-treatment start. Similar to 2D culture, CDT1 and p21 accumulation was detected in both cell lines. 

### 2.5. Efficacy of Pevonedistat In Vivo May Also Be Mediated by the Modulation of the Tumor Microenvironment in the Immune-Deficient Model

**Figure 5:**
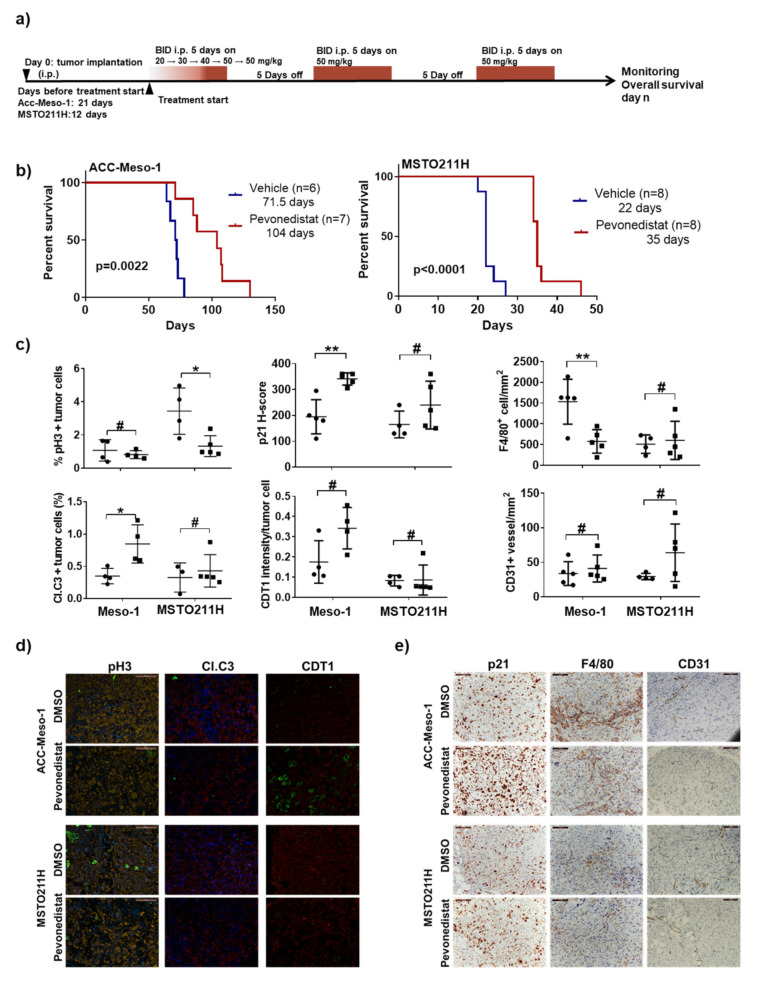
Efficacy and mechanisms of pevonedistat in an in vivo model. (**a**) Scheme depicting the schedule for tumor implantation and pevonedistat treatment cycles for the mouse model. (**b**) Kaplan-Meier plots showing significant differences in the overall survival (days) between the vehicle and pevonedistat-treated mice bearing an ACC-Meso-1 tumor (left) and MSTO211H tumor (right). (**c**) Comparison of tumor growth (pH3^+^), apoptosis (cleaved caspase 3, Cl.C3), CDT1 intensity, p21 H-score, mouse macrophage recruitment (F4/80^+^ cells per tumor area) and mouse vessel formation (CD31^+^ group of cells with visible lumen per tumor area) between vehicle-treated (filled circles) and pevonedistat-treated mice (filled squares). # not significant; * *p* < 0.05, ** *p* < 0.001, (**d**) Representative images of immunofluorescent staining for the indicated proteins with a green signal. They were co-stained with DAPI (blue) and pan-cytokeratin (yellow or red). The DAPI signal was omitted from CDT1 staining for a better visualization of the CDT1 signal. (**e**) Immunohistochemical staining of the indicated markers and counterstained with hematoxylin (blue). Scale bars represent 100 µm.

To further confirm the treatment efficacy and mechanism of action, we conducted in vivo experiments by selecting resistant and sensitive models. Among all cell lines tested, resistant ACC-Meso-1 and sensitive MSTO211H are tumorigenic in immunodeficient mice. We treated mice bearing tumors with pevonedistat with the dosage shown to be effective in other cancer models (50 mg/kg twice-daily (BID) applying a cycle of five days on and five days off the drug (Figure 5a)) [20]. MSTO211H tumor-bearing mice lost weight when treated with 50-mg/kg pevonedistat. Nevertheless, after the adaptation by receiving an increasing dosage during the first cycle, MSTO211H-bearing mice adapted to the treatment and tolerated the second cycle at 50-mg/kg BID (Figure S5). By monitoring animals closely daily, we used the overall survival as the readout for treatment efficacy. As expected, pevonedistat treatment prolonged the survival of MSTO211H-bearing mice by 13 days. Surprisingly, there was also a survival benefit of the treatment for ACC-Meso-1-bearing mice by 32.5 days (Figure 5b). The analysis of pevonedistat-treated tumor tissues showed a significant increase in p21 and CDT1 accumulation only in ACC-Meso-1-bearing mice (Figure 5c–e). Reduced tumor cell proliferation (pH3) was detected only in MSTO211H- but not ACC-Meso-1-bearing mice (Figure 5c,d). Surprisingly, we detected significantly increased apoptosis in an ACC-Meso-1-derived tumor (Figure 5c,d). ACC-Meso-1 is very resistant to pevonedistat and never underwent apoptosis, even with a high concentration of pevonedistat in 2D and 3D in-vitro models. We reasoned that there may be an effect of the treatment on the tumor microenvironment that induced apoptosis of the ACC-Meso-1 tumor in vivo. Thus, we analyzed cells associated with the tumor microenvironment, including mouse macrophage (F4/80+) and vessel formation (CD31+) that may explain the efficacy of pevonedistat in the ACC-Meso-1 model. At the baseline, ACC-Meso-1 tumors had a drastically higher macrophage infiltration (Figure 5c,e) compared to MSTO211H tumors. The treatment significantly reduced the numbers of tumor-associated macrophages in ACC-Meso-1 tumors, while it showed no effect on the MSTO211H tumors. There was no effect on the blood vessel formation in both tumor models at this time point (Figure 5c,e).

**Figure 6:**
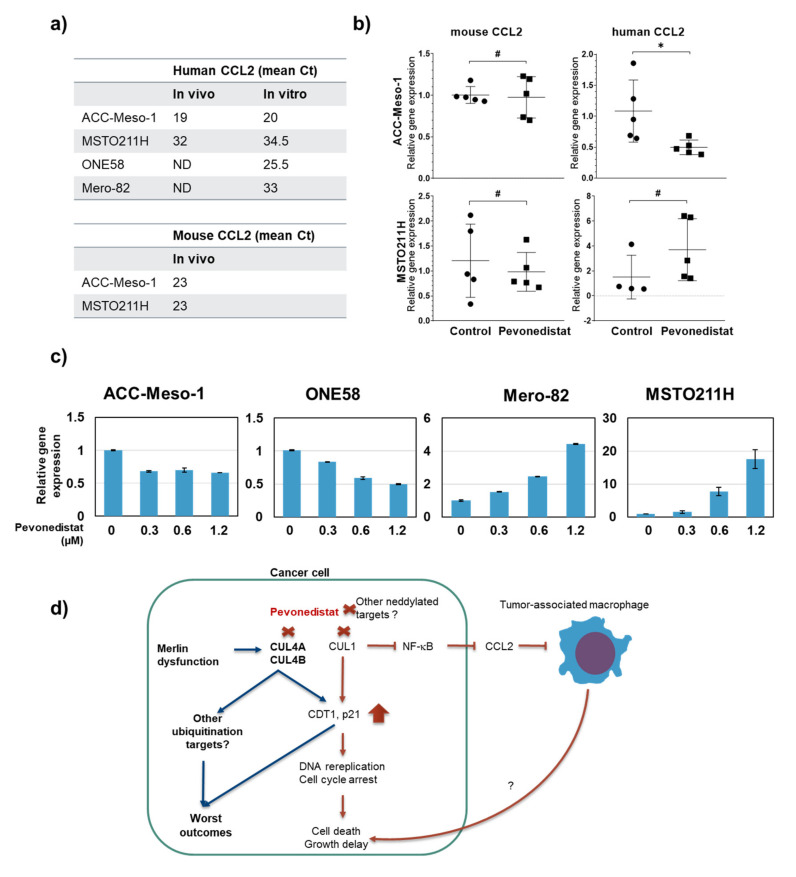
Effect of pevonedistat treatment on the expression of CCL2. (**a**) Comparison of the baseline expression levels of CCL2 in four different MPM cell lines in vitro. Lower Ct is equivalent to a higher expression. (**b**) qPCR analysis using mouse and human-specific CCL2 primers (using human-specific β-actin and mouse-specific β-2-microglobulin as a reference gene) showing a significant reduction of human CCL2 in the ACC-Meso-1 tumor treated with pevonedistat. (**c**) Change in the expression of human CCL2 in vitro after pevonedistat treatment in MPM cell lines. (**d**) Scheme illustrating the rationale and findings of our current study. CUL4A and CUL4B are shown to be regulated by Merlin dysfunction; here, we sought to investigate the importance of CUL4A and CUL4B expressions in MPM progression and tested the efficacy of pevonedistat, a small molecule inhibiting cullins via protein neddylation. We observed that a high CUL4B expression was associated with the worst clinical outcome. The treatment of MPM cells with pevonedistat resulted in an accumulation of CDT1 and p21, known targets of CUL4 and CUL1, leading to cell cycle arrest. Reduced nuclear factor- κB (NF-κB) pathway resulting from CUL1 inhibition caused a reduced CCL2 expression in tumor cells and reduced macrophage infiltration in tumors. Via an unknown mechanism, a reduced macrophage infiltration may result in more tumor cell death. It is important to note that CUL4A and CUL4B are shown to regulate several ubiquitination targets; thus, these targets may be important for MPM progression, and this remains to be investigated. In addition to cullins, pevonedistat also can affect some other proteins that are also regulated by neddylation. # not significant; * *p* < 0.05, Ct, threshold cycle and ND, not done.

An effect of pevonedistat on tumor-associated macrophage recruitment was shown in the lung cancer metastasis model, and the mechanism was mediated by a reduced expression of a macrophage chemotactic cytokine, C-C motif chemokine ligand 2 (CCL2), in tumor cells [21]. We therefore used mouse and human-specific primers to assess the expression of CCL2 in tumor tissues and found that the ACC-Meso-1 tumor expressed much higher levels of CCL2 at the baseline (approximately 16,000-fold higher than MSTO211H) (Figure 6a). The treatment significantly suppressed human CCL2 expression but had no effect on mouse CCL2 expression in the ACC-Meso-1 model (Figure 6b). There was no effect of the treatment on mouse CCL2 expression in the MSTO211H model. Although not significant, there seemed to be an increased expression of human CCL2 in the MSTO211H model (Figure 6b). To confirm this finding, we tested in vitro and included also two additional MPM cell lines in the analysis. The baseline expression of CCL2 was extremely different among those cell lines, being ACC-Meso-1 and ONE58 expressed high levels of CCL2 while Mero-82 and MSTO211H expressed very low-to-undetectable levels (Figure 6a). In agreement with the in vivo findings, an in vitro treatment with pevonedistat caused the downregulation of CCL2 in ACC-Meso-1 and ONE58. Interestingly, the opposite effect was detected in the Mero-82 and MSTO211H cell lines expressing low baseline levels of CCL2 (Figure 6c).

## 3. Discussion

Our results show that, in addition to being deregulated by *NF2* loss, a high expression of CUL4B ubiquitin ligase may be among the driving factors for MPM progression. Targeting cullins by the inhibition of protein neddylation is effective in 38% of MPM cell lines, while nonmalignant mesothelial cell lines are relatively resistant. Our data suggest that CUL4B may serve as an additional treatment target, and the inhibition of cullin activity using an inhibitor of protein neddylation may be efficacious for the treatment of MPM. We discovered that the pevonedistat treatment depleted tumor-associated macrophage infiltration (Figure 6d), which may be an additional favorable effect of pevonedistat for the treatment of cancers. 

We report here a significantly increased gene expression of CUL4A and, more significantly, CUL4B in a subset of patients with MPM compared to a population with an inflammatory disease of the pleura. Using publicly available data from TCGA, a high gene expression of CUL4B is associated with a worse disease-free survival of the patients in this cohort. Accordingly, our data on protein expression showed a significant association of CUL4B overexpression with worse clinical outcomes in MPM patients (Figure 6d). To our knowledge, this is the first report on an importance of CUL4B in MPM. Although sharing several similar functions with CUL4A, CUL4B harbors additional nuclear localization signals in the extended N-terminus. While CUL4B is strongly localized in the nucleus, CUL4A is mainly localized in the cytoplasm. CUL4B was shown to have unique roles in the nucleus, including histone modification and the epigenetic regulation of various target genes [22,23,24]. Thus, it can be hypothesized that they also possess independent cellular functions and, therefore, possess distinct roles in MPM. 

Using our cell line panel, we demonstrated that there was no correlation between gene and protein levels for CUL4A. This suggests that the expression of CUL4A in MPM is regulated by posttranslational modifications, in addition to the previously reported chromosomal gain [10]. It is still unknown why CUL4B is upregulated in MPM. Mutations of CUL4B located on chromosome Xq24 are associated with X-linked mental retardation syndrome, but its mutation has not been yet reported in cancers, including MPM. A recurring gain of this chromosomal region has also not been reported in MPM [25]. CUL4B has been shown to be regulated on the transcriptional level. A study in osteosarcoma identified tumor-suppressor miR-300 as a negative regulator for CUL4B expression [26]. Additionally, CUL4B expression was negatively regulated by miR-101-3p in lung cancer [27]. Although using a small-number cohort, miR-101-3p was shown to be downregulated in MPM compared to normal tissues [28]. Thus, it can be hypothesized that the upregulation of CUL4B in MPM is, in part, modulated by the alteration of miRNA levels and remains to be investigated.

There is a broad range of sensitivity of MPM cell lines in vitro to pevonedistat (IC_50_ ranging between 0.09–14.3 µM). This wide range of response has also been reported in a panel of melanoma and MPM cell lines [29,30]. It seems that the primary immediate effects of the pevonedistat treatment are caused by CDT1 accumulation, causing DNA rereplication and cell cycle arrest. Surprisingly, the treatment induced CDT1 accumulation in both sensitive and resistant cell lines, with no preferentiality towards the sensitive cell lines. Thus, there are other determining factors for the susceptibility of cells to CDT1 accumulation. One possible mechanism is the p53 pathway, as it has been shown that cell lines with p53 mutations are more susceptible to DNA rereplication compared to p53 wildtype after CDT1 overexpression [31]. p53 signaling was shown to be among the most significantly altered pathways in MPM [2]. p53 is mutated in about 8% of MPM patients [2] and thought to be a driver of the near-haploid subtype of MPM [25]. 

ACC-Meso-1 cells are very resistant to pevonedistat and only showed reduced proliferation after treatment with high concentrations in the 2D and 3D cultures. Nevertheless, in vivo, we observed increased apoptosis following the treatment without a growth delay. By observing cells associated with the tumor microenvironment, we found a strong reduction of the number of tumor-associated macrophages after pevonedistat treatment, which might be one of the reasons for the tumor growth delay detected in this model. This is in-line with the study using a lung cancer metastasis immunocompetent model that showed also reduced tumor-associated macrophages after pevonedistat treatment and, therefore, a reduced metastasis potential of tumor cells [21]. In the aforementioned immunocompetent model, it was shown that neddylation was important for monocyte chemotaxis to the tumor area, where they differentiated into tumor-associated macrophages, and this was accompanied by an increased number of tumor-infiltrating CD8^+^ T-cells. A reduced macrophage recruitment was mediated by a reduced chemotaxis factor CCL2 production of tumor cells resulting from a nuclear factor- κB (NF-κB) pathway blockade mediated by CUL1 inhibition. In our study, using human and mouse-specific primers, we also demonstrated reduced human CCL2 but not mouse CCL2 expression in vivo following the treatment with pevonedistat. Tumor-associated macrophages have been shown to play various roles in supporting tumor progression, such as promoting angiogenesis and the suppression of antitumor immunity [32]. Macrophages are abundantly present in MPM tumors, and it has been shown that the number of tumor-associated macrophages negatively correlates with the survival of patients with MPM sarcomatoid histology, supporting the tumor-promoting function of macrophages in MPM [33,34]. Moreover, the levels of CCL2 are elevated in the MPM pleural effusion on MPM patients compared to benign tissues [35], and CCL2 levels in the serum were elevated in late-stage MPM patients [36], suggesting their importance in MPM progression. Thus, the inhibition of CCL2 expression by pevonedistat may provide an additional benefit for MPM treatment, in addition to targeting only tumor cells.

Our investigation revealed the opposite effect of the treatment on the cell lines expressing low baseline levels of CCL2. The transcription of CCL2 was shown to be mainly dependent on the activity of NF-κB [37]; it can be speculated that NF-κB may be less active in these cell lines. One possible explanation of the increased CCL2 expression in these cells may be from an upregulation of exogenous stimuli. Nevertheless, the extent of increased CCL2 expression did not affect macrophage recruitment in this experimental model. 

In contrast to our in vitro findings, no increased apoptosis was detected in the MSTO211H in vivo model but only a suppression of tumor growth. MSTO211H formed aggressive fast-growing tumor nodules. Thus, due to the large-growing tumor volume, the drug may be poorly diffused into tumor nodules and cause only reduced proliferation in vivo. There is no effect of the treatment on tumor-associated macrophages in this model; this might be explained by the fact that there was much less tumor-associated macrophage recruitment in MSTO211H compared to ACC-Meso-1 tumors, which corresponded to a low baseline expression of CCL2 in MSTO211H in vivo and in vitro. 

Since our mouse model lacks both functional T cells and B cells, it can be assumed that the effects on tumor growth we have seen here are purely mediated by macrophage depletion. Tumor-suppressing effects of pevonedistat may be more pronounced when functional T cells are present, and this remains to be investigated.

## 4. Materials and Methods

### 4.1. MPM Tissue Microarrays (TMAs)

Tumor samples from 190 MPM patients were collected between 1999 and 2009. Patient’s characteristics and treatment are described in Table S1. The study was approved, and a waiver of consent was granted by the Ethical Committee Zürich (StV 29-2009 and EK-ZH 2012-0094). 

### 4.2. Immunohistochemical Staining and Quantification for Protein Expression

Two-micrometer-TMA or tissue sections were deparaffinized and rehydrated. The staining for CUL4A (LSBio, LS-B360, Seattle, WA, USA), CUL4B (Sigma, HPA HPA011880, Buchs, Switzerland), p21 (Abcam, ab109199, Cambridge, UK), F4/80 (BMA Biomedicals, Augst, Switzerland) and CD31 (Abcam, ab28364, Cambridge, UK) was performed with a Dako Autostainer Link48 Instrument (Dako Denmark A/S) according to the manufacture’s instructions. Antigen retrieval were achieved using target retrieval solution pH 6 (Dako, K8005, Agilent Dako, Basel, Switzerland) for CUL4A and F4/80 and pH 9 (Dako, K8004, Agilent Dako, Basel, Switzerland) for CUL4B, CD31 and p21. We used a visualization system consisting of the Dako EnVision™ rabbit/HRP/DAB system and counterstained with hematoxylin. CUL4B antibody was developed and validated by the Human Protein Atlas (HPA) project. We tested the specificity of immunohistochemical staining of the CUL4A antibody using cell blocks from Met5A (low CUL4A expression) and Mero-82 (high CUL4A expression) and observed a concordance between protein expressions tested by Western blotting (Figure 2c) and immunohistochemical staining (Figure S6). Immunohistochemical staining score for TMAs (H-score) from scanned images was obtained by a multiplication of intensity (0–3) with the staining frequency (0–1) [7] in a blind fashion. M.M., J.K. and V.O. conducted CUL4A and CUL4B scoring on TMAs. M.M evaluated p21 from the in vivo experiment using a modified H-scoring system, while the intensity was scored as for TMAs (0–3); the frequency of cells with each staining intensity was estimated as an actual % of the total cells (1–100%). Total H-score (ranging from 0–400) is the sum of each staining intensity multiply by their frequency. Numbers of F4/80-positive cells and blood vessels (CD31+) were normalized with the tumor area (mm^2^). 

### 4.3. Tissue Samples for RNA Extraction

Tissue samples from 75 MPM patients and 18 patients with inflammatory disease of the pleura were collected at MPM diagnosis, surgery or relapse. All patients gave inform consent, and the study was approved by the Ethical Committee Zürich (ethical approval number: EK-ZH 2015-2171). After collection, tissues were immediately placed in RNAlater solution (Qiagen, Hombrechtikon, Switzerland) for preservation of the RNA integrity. Fresh tumor tissues harvested from the in vivo experiment were immediately placed in liquid nitrogen and stored at −80 °C until analysis.

### 4.4. RNA Extraction, cDNA Synthesis and Quantitative Real-Time PCR

We employed a TRIzol reagent (Invitrogen 15596018, Life Technology Europe BV, Zug, Switzerland), using the protocol suggested by the manufacturer, for RNA extraction. We used PrimeScript RT with gDNA Eraser (TAKARA Bio, S-86901-06-01, Saint-Germain-en-Laye, France,) for cDNA synthesis and KAPA SYBR FAST qPCR master mix with ROX (Kapa Biosystems, KK4621 Merck, Buchs, Switzerland) for quantitative real-time PCR with a 7500 real-time PCR system (Life technology, Life Technology Europe BV, Zug, Switzerland). For MPM, only specimens containing >50% tumor contents were selected for the analysis. We estimated tumor contents by analyzing gene expressions of calretinin, mesothelin and podoplanin normalized against histone RNA, as previously described [38]. A list of primers is provided in Table S3.

### 4.5. Cell Lines

Mero-83, Mero-84, Mero-41, Mero-14, Mero-25, Mero48a, Mero-82, NO36 and ONE58 were obtained from the European Collection of Cell Cultures (Salisbury, UK). ACC-Meso-1 and ACC Meso-4 were purchased from Riken BRC (Ibaraki, Japan). Met5A and MSTO211H cell lines were from ATCC (Wesel, Germany). Dr. Emanuela Felley-Bosco kindly provided ZL55 and SDM104 [18]. All cell lines were maintained in RPMI1640 with ATCC modification (Gibco, A10491-01 Life Technology Europe BV, Zug, Switzerland,), supplemented with 10% fetal bovine serum (FBS) and penicillin/streptomycin (p/s). All cell lines were regularly tested for mycoplasma contamination.

### 4.6. Treatment and Cell Survival Analysis for 2D and 3D Culture

For 2D cultures, cell lines were seeded with 3000 cells/well in a 96-well plate. Twenty-four hours later, cells were treated with different concentrations of pevonedistat (2-fold dilutions, ranging from 0.039 to 5 µM) in their complete medium. At 72 h after the treatment, cell survival was measured by 3-(4,5-dimethylthiazol-2-yl)2,5-diphenyltetrazolium bromide (MTT) assay. Briefly, cells were exposed to 1-mg/mL MTT solution (3-(4,5-dimethylthiazol-2-yl)2,5-diphenyltetrazolium bromide) in Dulbecco’s modified Eagle’s medium (DMEM)/F12 medium without phenol red plus 0.5% FBS for 90 min, followed by cell lysis in a solution containing 50% dimethylformamide (DMF) and 20% sodium dodecyl sulfate (SDS) pH 4.7 and optical density (OD) measurement at 570 nm.

### 4.7. Protein Extraction and Western Blot

We used radioimmunoprecipitation assay (RIPA) buffer containing a proteinase and phosphatase inhibitor cocktail (Cell Signaling, #5872, Danvers, MA, USA) for cell lysis for 20 min, followed by DNA shearing by sonication. The following antibodies were used for Western blot experiments: CUL4A, Cell Signaling, Danvers, MA, USA, CUL4B, Sigma HPA011880, Buchs, Switzerland, actin, Santa Cruz I19, Heidleberg, Germany, tubulin, Abcam ab6046 Cambridge, UK, CDT1, Cell Signaling, Danvers, MA, USA, p21 BD Bioscience, San Jose, CA, USA, cleaved caspase-3, Cell Signaling, Danvers, MA, USA, and phospho-histone H3 (Ser10) (pH3), Cell signaling, Danvers, MA, USA. Western blot imaging and signal quantification was performed using Fusion FX7 (Witec AG, Sursee, Switzerland). Uncropped blots with molecular weight markers are provided as Figure S7.

### 4.8. Cell Cycle Analysis

The treatment with pevonedistat was performed in a cell culture medium for 24 h. Following fixation with methanol, cells were permeabilized with 0.25% TritonX-100 and subjected to staining with anti-pH3 antibody (Cell Signaling, Danvers, MA, USA; diluted 1:50 in 5% bovine serum albumin (BSA)/0.2% Triton/phosphate-buffered saline (PBS)). Secondary antibody was goat anti-rabbit Alexa488-conjugated antibody (Invitrogen, A11034, Life Technology Europe BV, Zug, Switzerland), followed by co-staining with propidium iodide (PI). Data were acquired by a flow cytometer (Attune, Applied Biosystems, Life Technology Europe BV, Zug, Switzerland) and analyzed by FlowJo software v.10 (BD Biosciences, San Jose, CA, USA).

### 4.9. MPM Spheriod Cultivation, Treatment and Viability Measurement

To prepare a nonadherent concaved surface for spheroid formation, 96-well cell culture plates were filled with 50-µL melted sterile agarose. A different starting cell seeding number in RPMI 1640 with ATCC modification supplemented with 10% FBS and (P/S) was used to achieve a spheroid diameter of 370–400 µm on day 4 after seeding [39]. Spheroids were treated with different concentrations of pevonedistat (2-fold dilution ranging from 0.312 to 2.5 µM) or cisplatin (2-fold dilution ranging from 2.5 to 20 µM) in the full culture medium on day 4 after seeding and refreshed on day 7. We monitored the drug efficacy by measuring the spheroid diameter on days 4 (before the treatment), 7, 9 and 11 (Axiovert 40 CFL (Zeiss, Oberkochen, Germany)). At the endpoint on day 11, we measured the cell viability by acid phosphatase (APH) assay [40]. Briefly, spheroids were washed with Dulbecco’s phosphate-buffered saline DPBS; afterwards, 100 µL of 2-mg/mL p-nitrophenyl phosphate, disodium salt, hexahydrate (PNPP) (Thermo scientific, 34045, Life Technology Europe BV, Zug, Switzerland) in 0.1-M sodium acetate, 0.1% Triton X-100/water were added to a spheroid in 100-µL D-PBS. After 90 min incubation, we added 10-µL 1-N NaOH to stop the enzymatic reaction. The change in pH to alkaline resulted in p-nitrophenol turning yellow, which was measured at 405 nm within 10 min.

### 4.10. Immunofluorescence

Frozen tumor tissues or spheroids embedded in optimal cutting temperature (OCT) medium were cryosectioned into 10 µm and air-dried. For CDT1 staining, slides were fixed in Delaunay fixative and air-dried. Slides were rehydrated, fixed with 4% paraformaldehyde, permeabilized with 0.2% triton X-100 in PBS and blocked with 4% BSA, 5% normal serum and 0.1% Tween in PBS. We used the following antibodies diluted in 2% BSA, 2.5% normal serum in PBS, 0.1% Tween: pH3 (Abcam, ab5176, Cambridge, UK, 1:200), Cl.C3 (Cell signaling, Danvers, MA, USA, 1:400), pan-cytokeratin (Abcam, ab86734, Cambridge, UK, 1:300), CDT1 (Abcam, Ab202067, Cambridge, UK, 1:50), goat anti-rabbit IgG Alexa 488 (Thermo Scientific, A11008, Life Technology Europe BV, Zug, Switzerland, 1:500) and donkey anti-mouse IgG Alexa 555 (Thermo Scientific, A31570, Life Technology Europe BV, Zug, Switzerland, 1:500). We used an additional blocking step with anti-mouse IgG for the staining on mouse tissues (Thermo Scientific, A16074, Life Technology Europe BV, Zug, Switzerland, 1:25). Images were acquired by a Leica DM6000 (Leica Microsystems, Heerbrugg, Switzerland). We counted tumor cells (pan-cytokeratin+) positive for pH3 and Cl.C3 manually, and all tumor cells were quantified for CDT1 intensity using Image J 1.52a (Madison, WI, USA) software in at least 900 tumor cells. 

### 4.11. Animal Experiment

The animal experiment was performed according to the regulation of the Veterinary Office of the Canton, Zurich, Switzerland. Six-to-8-week-old male Fox Chase severe combined immunodeficiency (SCID) mice (CB17/Icr-Prkdc^scid^/IcrIcoCrl; IcrIcoCrl, Charles River, Sulzfeld, Germany) were housed according to the guidelines for animal welfare. We randomized mice into 4 groups (ACC-Meso-1 control and -treated, and MSTO211H control and -treated) after at least 2 weeks’ adaptation. Tumor implantation by intraperitoneal injection (i.p.) was performed on day 0 (21 days before the treatment start for mice receiving Meso-1 and 12 days before the treatment start for MSTO211H). We implanted 5 × 10^6^ cells ACC-Meso-1 or 1 × 10^6^ cells MSTO211H in 200-µL sterile DPBS. Both cell lines were authenticated and tested to be free from rodent and human pathogens, as well as mycoplasma. Pevonedistat (Active Biochem, Kowloon, Hong Kong) was freshly prepared in sterile 3.33% dimethylsulfoxide (DMSO) and 20% cyclodextrin in water with a concentration of 10 mg/mL. Mice were treated twice-daily (BID) i.p. with pevonedistat for 3 cycles of 5 days’ treatment with 5 days’ treatment-free period; see Figure 5a. We had to increase the dosage gradually during the first treatment cycle due to weight loss of MSTO211H tumor-bearing mice if they were immediately treated with a high dose. Administration of a high dose in the second and third cycles was tolerated in both groups (see body weight changes in Figure S5). Mice were monitored daily and blindly after tumor implantation until the end of the experiment for signs of pain and distress, changes in activity, body weight, body condition score [41] and ascites formation. In case of impaired health status measured by the previously mentioned criteria, they were humanely euthanized, and the time from tumor cell implantation to time of euthanasia was considered as the overall survival. To analyze the changes in tissues following the treatment, mice were treated with 1 cycle of pevonedistat; tumor tissues were excised and immediately preserved for further analyses.

### 4.12. Statistical Analyses

We used SPSS v.25 (IBM, New York, NY, USA) for survival analysis of the MPM patient cohort and GraphPad Prism v.8.0.0 (San Diego, CA, USA) for Mann Whitney, *t*-tests, IC_50_ calculation and survival analysis of the in vivo experiments.

## 5. Conclusions

Although a relatively rare disease, MPM is fatal and difficult to treat. The standard of care treatment still relies on DNA-damaging agents and antimetabolite-like cisplatin and pemetrexed. Although the lack of oncogenic driver hinders the development of targeted treatment for MPM, deciphering pathway alterations resulting from tumor-suppressor gene loss may shed light on additional key molecules that can serve as a treatment target for MPM. Previous studies have shown that CUL4 ubiquitin ligase complex is one of the pathways being unleashed when the key tumor suppressor for MPM, *NF2*, is mutated. Here, we discovered that CUL4 paralogs CUL4A and CUL4B were also upregulated in MPM and a high expression of CUL4B correlated with the worst prognosis, underlying the importance of CUL4 ubiquitin ligase in MPM. Thus, alterations of the ubiquitination event may be one of the key factors driving mesothelioma, and this is emphasized by the fact that another tumor suppressor frequently mutated in MPM, BRCA1-associated protein-1 (*BAP1)*, also functions as a nuclear deubiquitinating enzyme. Thus, it is important to investigate in more detail regarding the mechanisms of CUL4B upregulation and how the overexpression drives MPM progression. A subset of MPM cell lines are sensitive to pevonedistat, a small molecule that inhibits all cullins by targeting protein neddylation. Interestingly, the treatment also induced a tumor response by targeting the immune microenvironment, which may provide additional favorable clinical benefits. Nevertheless, pevonedistat has a broad spectrum of targets, as all cullins and some other proteins like p53 are also regulated by neddylation. Therefore, identifying a targeted agent that is specifically acting on CUL4B or its complex may further improve the clinical benefits and minimize the undesirable side effects. 

## Supplementary Materials

The following are available online at https://www.mdpi.com/2072-6694/12/11/3460/s1: Figure S1: IC_50_ curves of all cell lines. Figure S2: Cell cycle analysis by PI staining showing increased S or G2/M populations or cells containing >4N in all cell lines tested. Figure S3: The impact of cisplatin on the sphere volume of the four cell lines over the treatment time of eleven days. Figure S4: Dose response and IC_50_ of the spheroids from the four cell lines to cisplatin. Figure S5: Changes in body weight compared to day 0 (before tumor implantation) of mice bearing a MSTO211H tumor. Figure S6: Immunohistochemical staining of CUL4A in cell blocks generated from Met5A and Mero-82. Figure S7: Uncropped blots with molecular weight markers. Table S1: Characteristics of the patient cohort employed to assess the protein expressions of CUL4A and CUL4B on tissue microarrays in Figure 1b. TableS2: IC_50_ of pevonedistat in cell lines depicted in Figure 2a. Table S3: List of primers for SYBR quantitative real-time PCR.
