# Supplementary material for: Importance of Cullin4 Ubiquitin Ligase in Malignant Pleural Mesothelioma
Source: Cancers (Basel). 2020 Nov 20;12(11):3460. doi: 10.3390/cancers12113460 (PMC7699720; doi:10.3390/cancers12113460)
Supplement: Supplementary file 1 [file cancers-12-03460-s001.pdf]

cancers

MDPI

Article

Importance of Cullin4 Ubiquitin Ligase in Malignant Pleural Mesothelioma

Mayura Meerang *, Jessica Kreienbühl, Vanessa Orlowski, Seraina L. C. Müller, Michaela B. Kirschner and Isabelle Opitz *

Department of Thoracic Surgery, University Hospital Zürich, Zürich, 8091, Switzerland; Jessica.Kreienbuehl@usz.ch (J.K.); Vanessa.Orlowski@usz.ch (V.O.); seraina.bomue@hotmail.com (S.L.C.M.); Michaela.Kirschner@usz.ch (M.B.K.)

* Correspondence: Mayura.Meerang@usz.ch (M.M.); Isabelle.Schmitt-Opitz@usz.ch (I.O.)

Received: date; Accepted: date; Published: date

Supplementary

Table S1. Characteristics of the patient cohort employed to assess protein expression of CUL4A and CUL4B on tissue microarrays in figure 1b.

Parameter	Number of cases (%)
**Age** (year) (median; range)	61.5 (35.8-81.0)
**Gender**	
Male	129 (90.2)
Female	14 (9.8)
**Treatment**	
Ind. CTX followed by extrapleural pneumonectomy	81 (56.6)
Ind. CTX & pleurectomy/decortication	1 (0.7)
Ind. CTX & Palliative surgery	12 (8.4)
Ind. CTX & no surgery	14 (9.8)
Palliative CTX	28 (19.6)
Best supportive care	7 (4.9)
**Histology**	
Epithelioid	91 (63.6)
Biphasic	42 (29.4)
Sarcomatoid	8 (5.6)
Data missing	2 (1.4)
**IMIG Stage**	
I	9 (6.3)
II	23 (16.1)
III	53 (37.1)
IV	24 (16.8)
Data missing	34 (23.8)

Ind. CTX, induction chemotherapy.

Table S2. IC50 of pevonedistat in cell lines depicted in figure 2a.

	N	Mean	Std. Deviation
**Mero-83**	3	14.29	3.47
**Mero-84**	3	9.17	0.94
**ACC-Meso-4**	2	5.60	0.85
**SDM104**	2	2.83	1.52
**Mero-41**	3	2.90	1.61
**MET5A**	18	2.86	0.49
**ACC-Meso-1**	3	1.35	0.44
**ZL55**	3	0.78	0.22
**Mero48a**	3	1.00	0.26
**NO36**	3	0.93	0.38
**Mero-25**	3	0.46	0.09
**ONE58**	4	0.26	0.13
**Mero-14**	3	0.18	0.04

**Mero-82**	4	0.12	0.05
**MSTO211H**	2	0.06	0.01

Table S3. List of primers for SYBR quantitative real time PCR.

Gene	Primer sequence (5'-3')
Human CUL4A	FW: CAGGCACAGATCCTTCCGTT<br>RE: TCCTGCCAGCACGTGTTAAT
Human CUL4B	FW: GGACATGGGACTGGAGTTATTT<br>RE: ACCATTCCTTTCCCTCTCAATC
human Beta-Actin (ACTB)	FW: GGACCTGACTGACTACCTCAT<br>RE: CGTAGCACAGCTTCTCCTTAAT
Human Calretinin	FW: GGATAGAAGCGGCTACATTGAC<br>RE: CGCTCTTTCTGTAGTTGGTGAG
Human Podoplanin	FW: GACACTGAGACTACAGGTTTGG<br>RE: GCCAGACTTATAGCGGTCTTC
Human Mesothelin	FW: AACATGAACGGGTCCGAATAC<br>RE: CCAAGTCCATGCTCACATTCT
Human Histone H3 [1]	FW: GGTAAAGCACCCAGGAAGCA<br>RE: CCTCCAGTAGAGGGCGCAC
human specific CCL2 (Primer Bank ID:4506841a1) [2]	FW: CAGCCAGATGCAATCAATGCC<br>RE: TGGAATCCTGAACCCACTTCT
human specific Beta-actin (ACTB)	FW: CCTCGCCTTTGCCGATCC<br>RE: CGCGGCGATATCATCATCC
mouse specific CCL2 (Primer Bank ID:141803162c1) [2]	FW: TTAAAAACCTGGATCGGAACCAA<br>RE: GCATTAGCTTCAGATTTACGGGT
mouse specific β-2-microglobulin (B2M)	FW: GGTCTTTCTGGTGCTTGTCT<br>RE: TATGTTCGGCTTCCCATTCTC

1. Andre, M.; Felley-Bosco, E. Heme oxygenase-1 induction by endogenous nitric oxide: Influence of intracellular glutathione. *FEBS letters* **2003**, *546*, 223-227.

2. Wang, X.; Seed, B. A pcr primer bank for quantitative gene expression analysis. *Nucleic Acids Res* **2003**, *31*, e154.

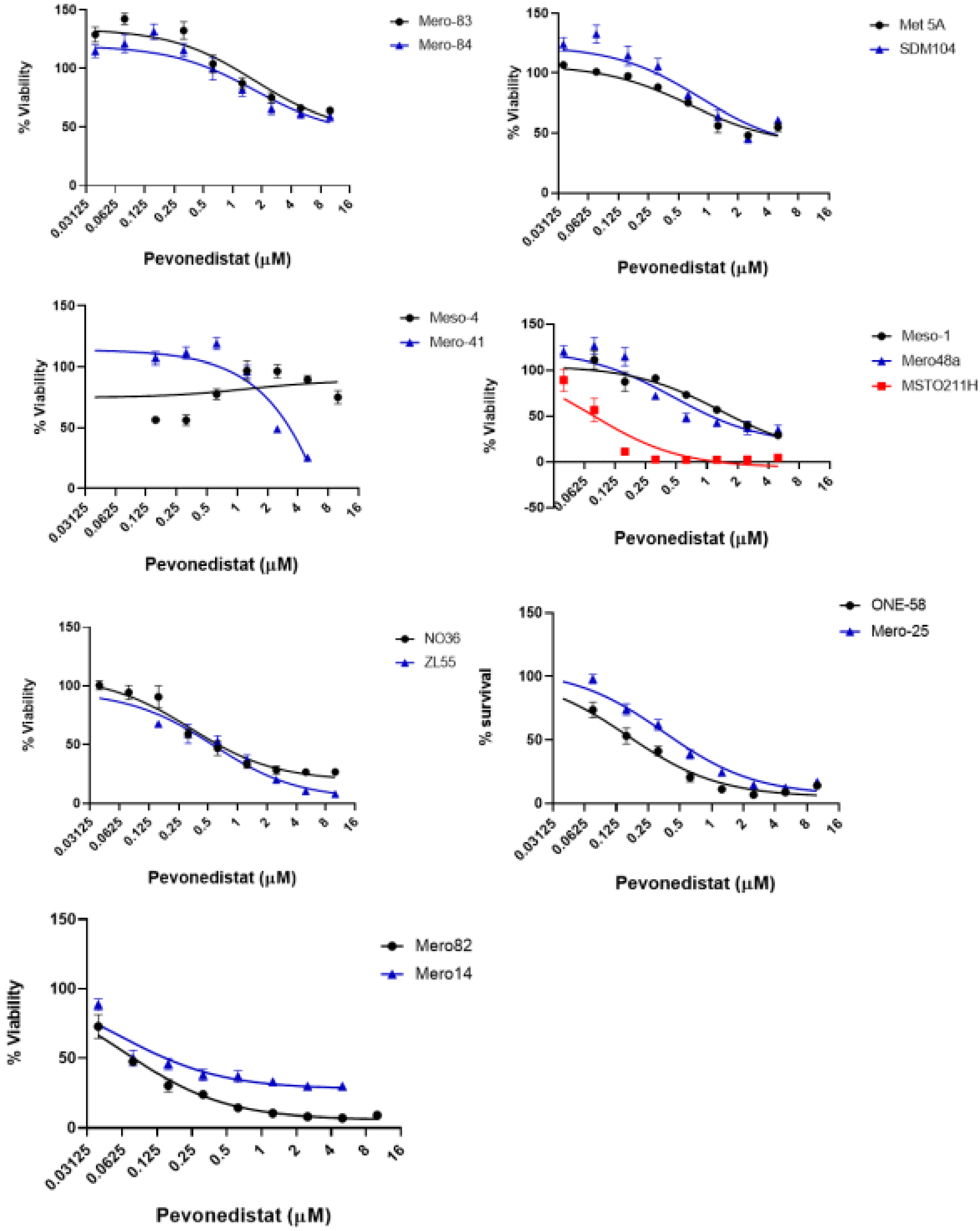

Figure S1. IC50 curves of all cell lines.

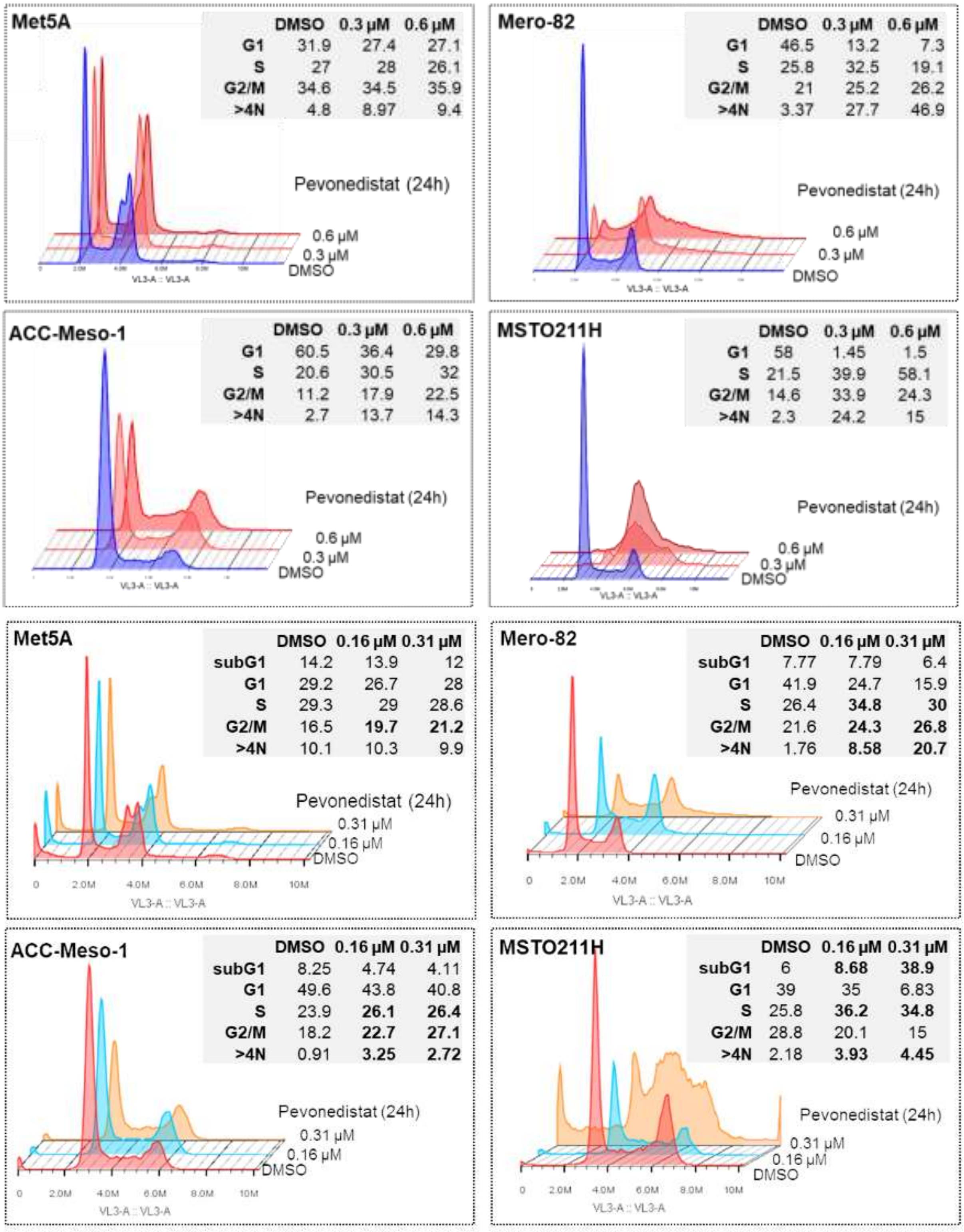

Figure S2. Cell cycle analysis by PI staining showing increased S or G2/M pupulations or cells containing >4N in all cell lines tested.

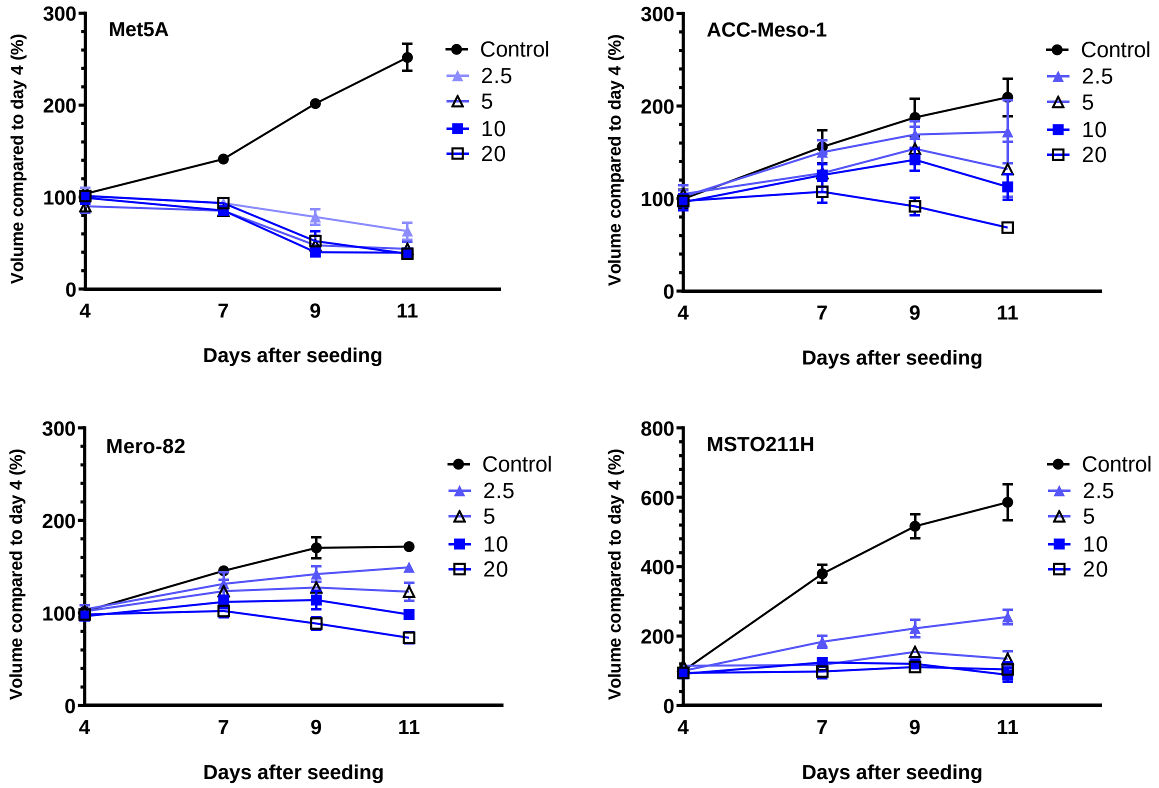

Figure S3. The impact of cisplatin (μM) on the sphere volume of the four cell lines over the treatment time of eleven days.

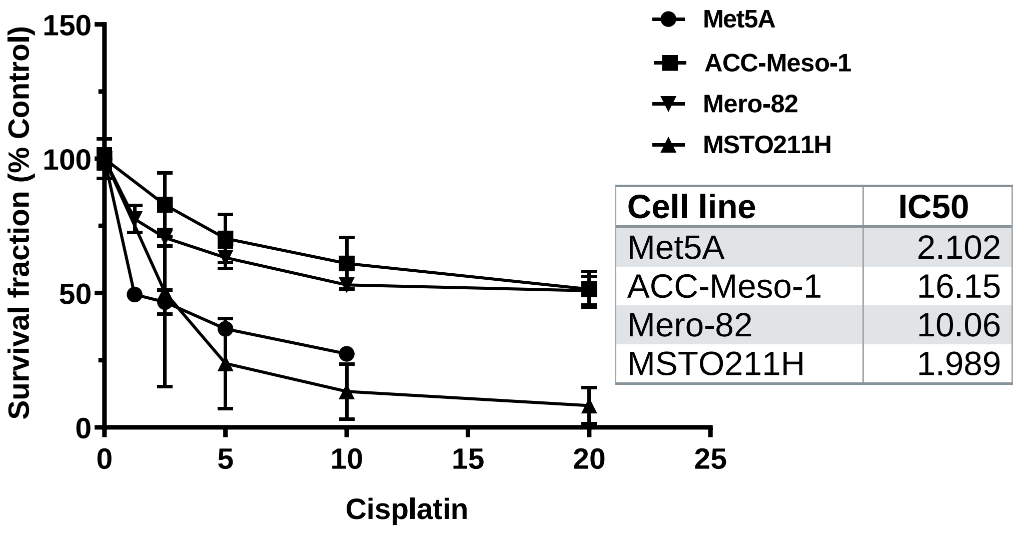

Cell line	IC50
Met5A	2.102
ACC-Meso-1	16.15
Mero-82	10.06
MSTO211H	1.989

Figure S4. Dose response and IC50 (μM) of the spheriods from four cell lines to cisplatin.

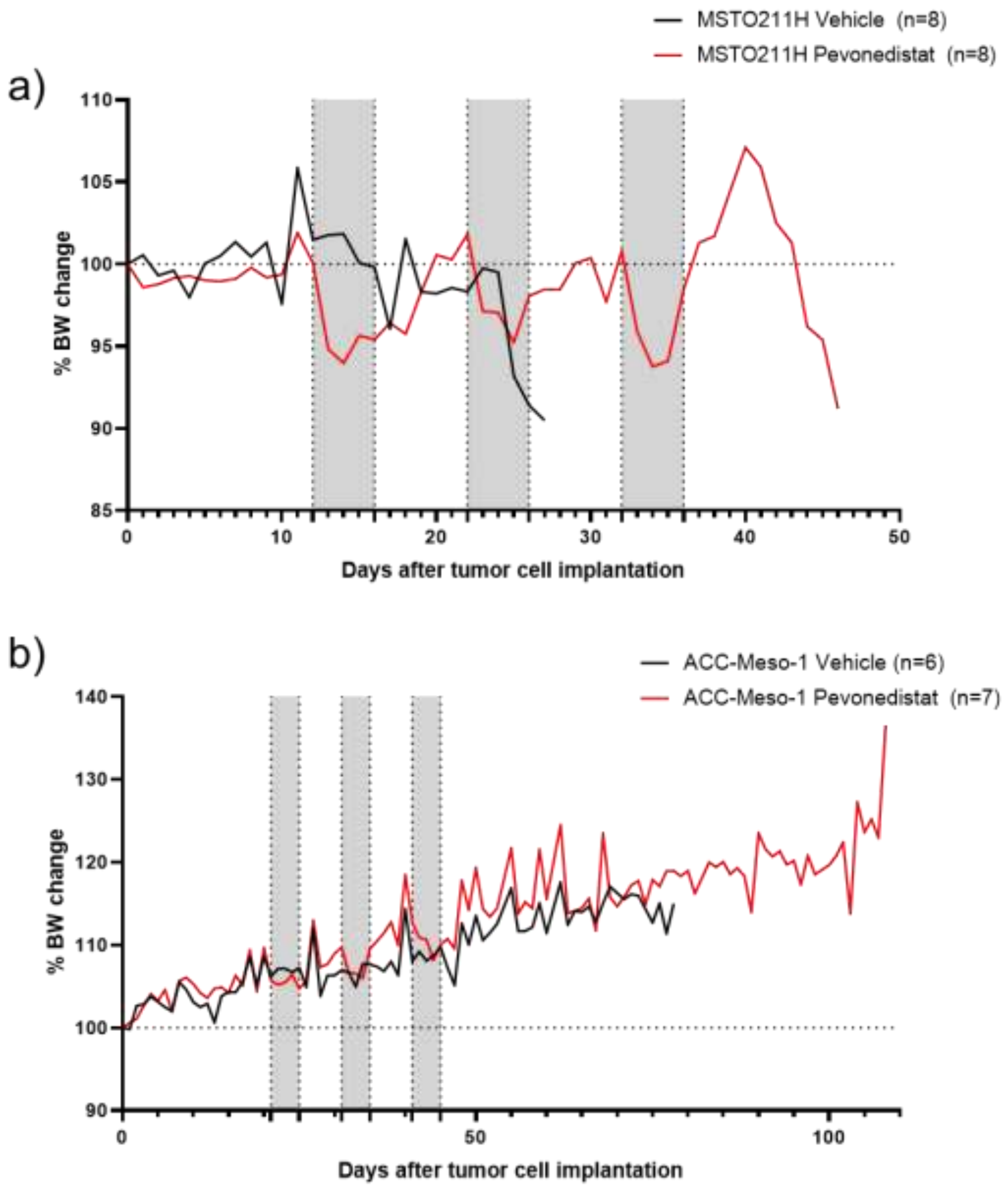

Figure S5. Changes in body weight compared to day 0 (before tumor implantation) of mice bearing MSTO211H tumor (a) or ACC-Meso-1 tumor (b). Three treatment cycles (5 days treatment) are highlighted in gray. Weight loss occurs in both vehicle and pevonedistat treated groups during the treament period and recovered during treatment free period. MSTO211H baring mice are more affected by the treatment during the first treatment cycle. Data are presented as mean body weight loss from all amimals in the group.

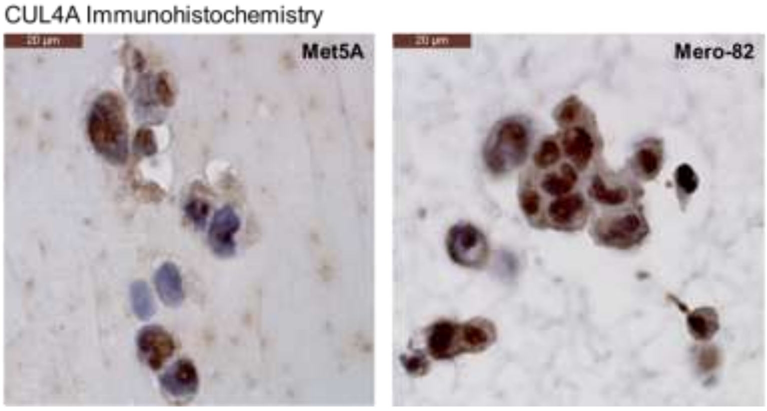

Figure S6. Immunohistochemical staining of CUL4A in cell blocks generated from Met5A and Mero-82.

Figure 2c

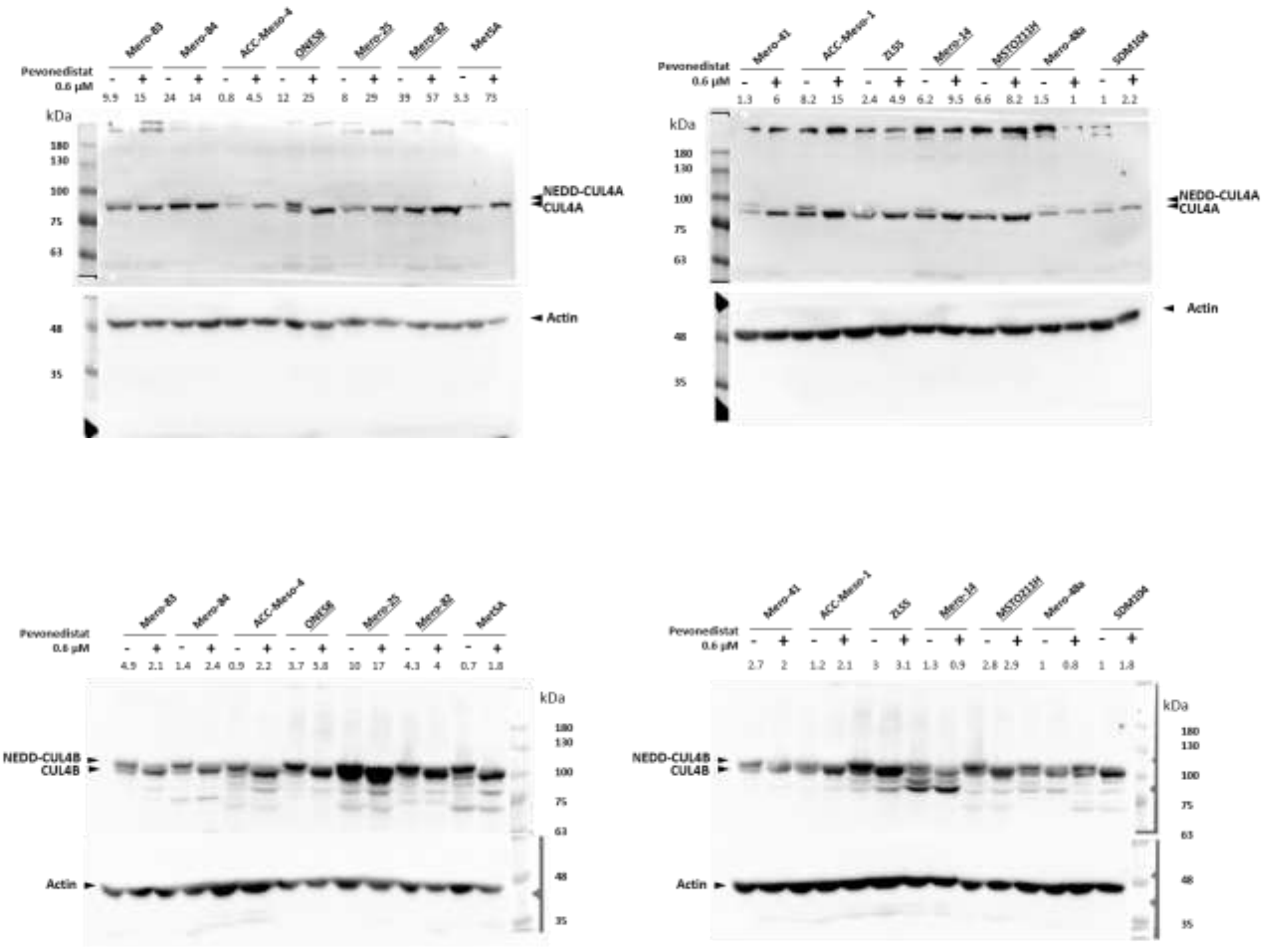

Figure 3b

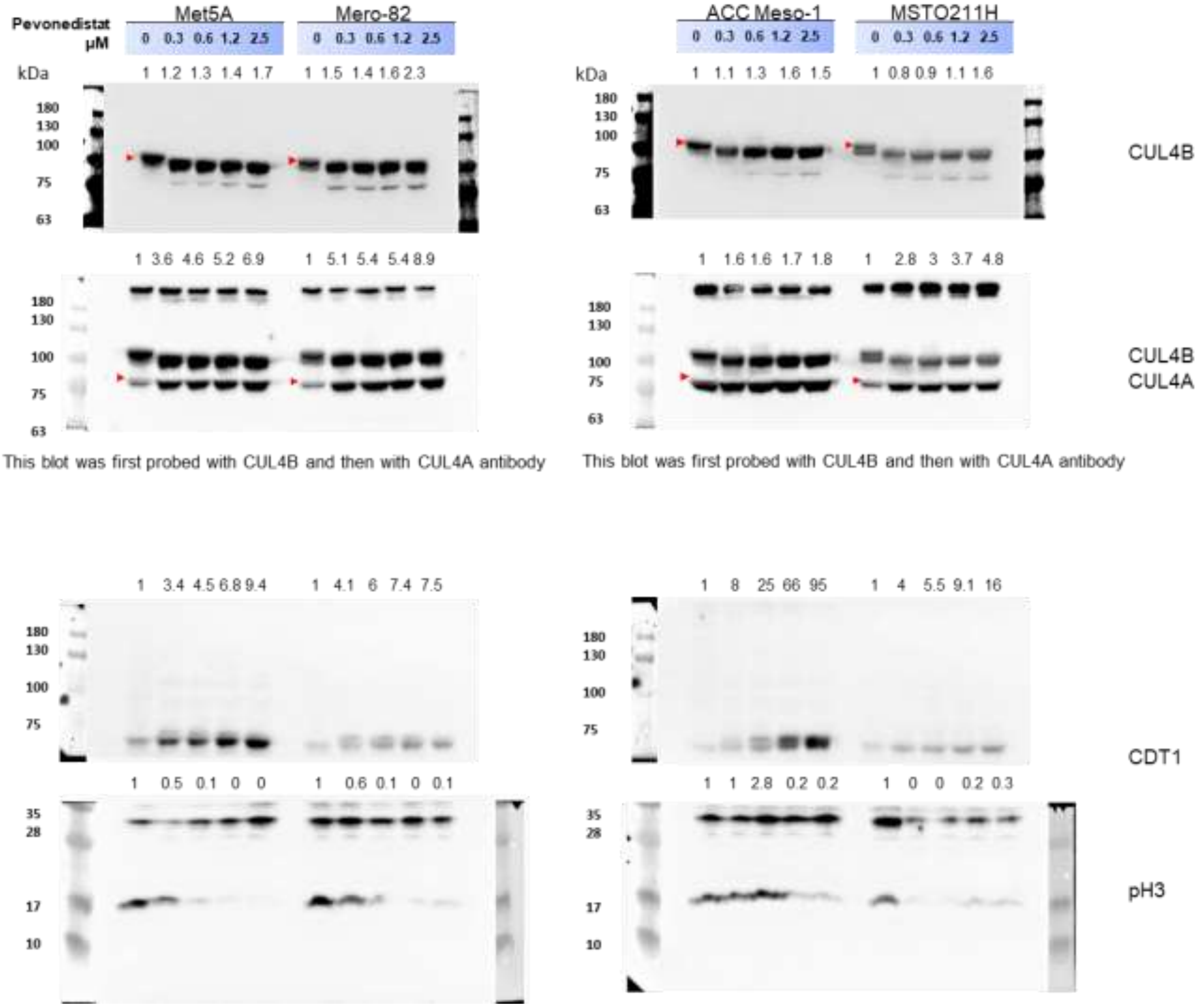

Pevonedistat μM
Met5A
Mero-82
ACC Meso-1
MSTO211H
0 0.3 0.6 1.2 2.5
kDa
1 1.2 1.3 1.4 1.7
1 1.5 1.4 1.6 2.3
1 1.1 1.3 1.6 1.5
1 0.8 0.9 1.1 1.6
CUL4B
1 3.6 4.6 5.2 6.9
1 5.1 5.4 5.4 8.9
1 1.6 1.6 1.7 1.8
1 2.8 3 3.7 4.8
CUL4B
CUL4A
This blot was first probed with CUL4B and then with CUL4A antibody
1 3.4 4.5 6.8 9.4
1 4.1 6 7.4 7.5
1 8 25 66 95
1 4 5.5 9.1 16
CDT1
1 0.5 0.1 0 0
1 0.6 0.1 0 0.1
1 1 2.8 0.2 0.2
1 0 0 0.2 0.3
pH3

Figure 3b cont.

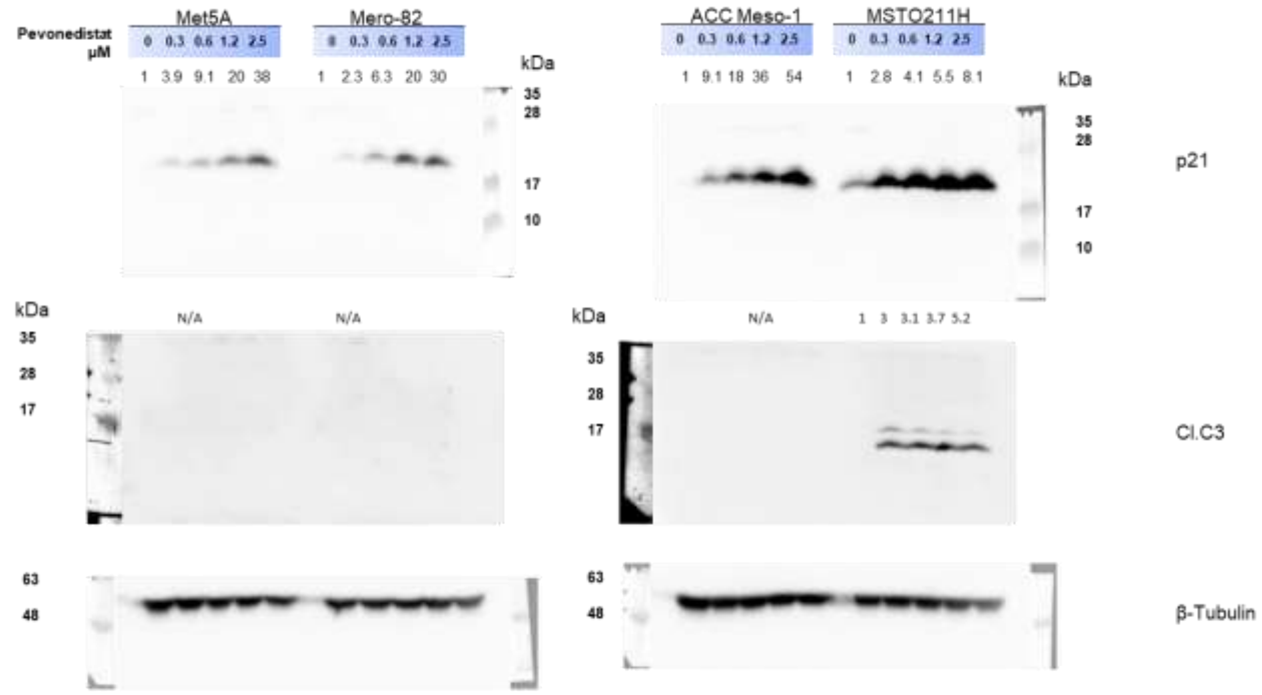

Figure 3c

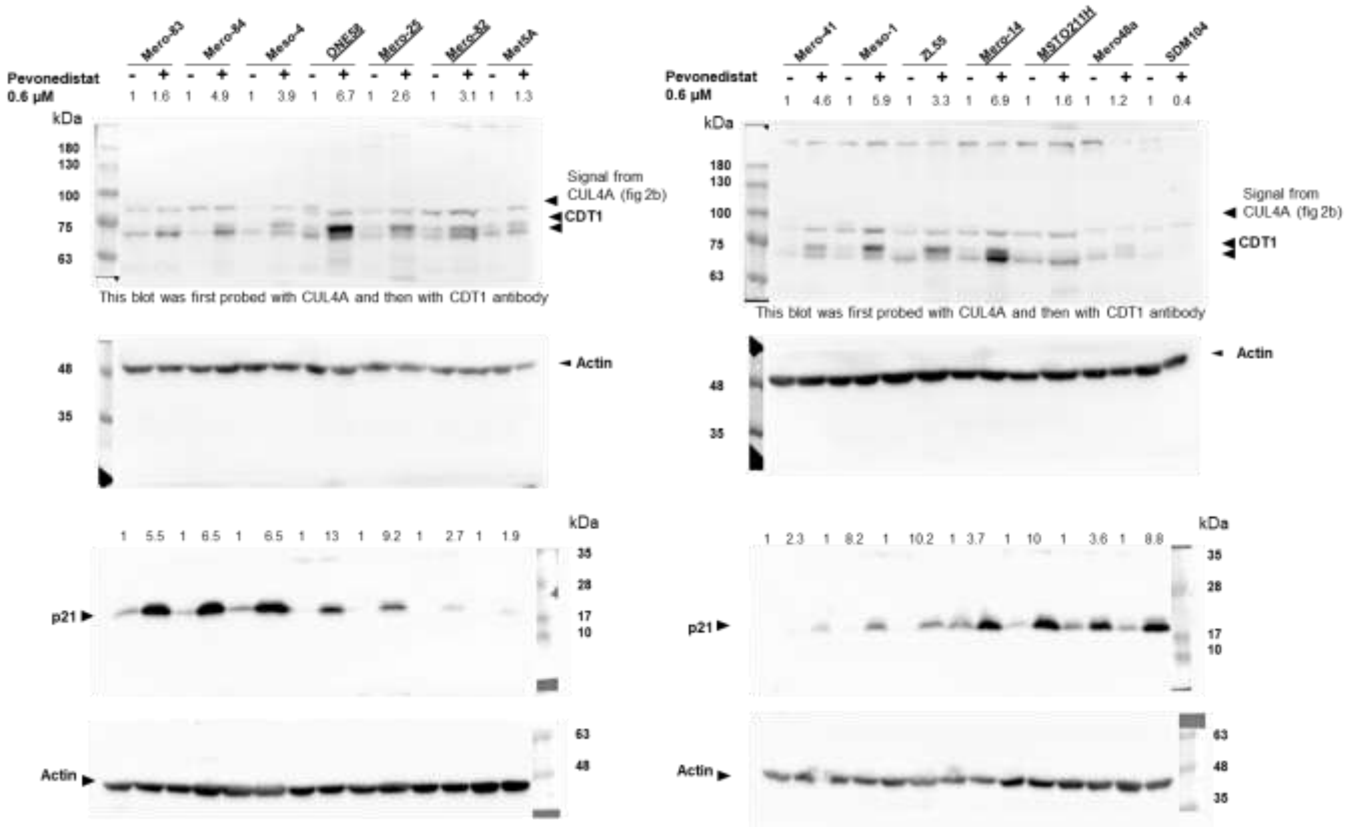

Figure S7. Uncropped blots with molecular weight markers from figure 2c, 2d, 3b, 3c in the main manuscript.
